# Supplementary material for: Are carbon emissions trading and green financial instruments synergistic? -Comprehensive quantitative research based on content analysis
Source: PLoS One. 2024 Mar 7;19(3):e0298601. doi: 10.1371/journal.pone.0298601 (PMC10919601; doi:10.1371/journal.pone.0298601)
Supplement: S1 Data — (ZIP) [file pone.0298601.s001.zip › Data/地级市Z_F工作报告.zip!/╒π╜¡/║╝╓▌╩╨/└┤╫╘╣1⁄2╓┌║┼í╛┬φ┐╦╩2╛▌═°í┐_2015.docx]

各位代表：

　　现在，我代表市人民政府向大会作政府工作报告，请予审议，并请市政协委员和其他列席人员提出意见。

　　一、2014年主要工作

　　2014年是全面深化改革的开局之年。一年来，市政府认真贯彻落实党中央、国务院，省委、省政府和市委各项决策部署，全面执行市十二届人大四次会议决议，紧紧依靠全市人民，全力稳增长、调结构、抓改革、强统筹、治环境、惠民生、促和谐，较好实现了年度目标任务。全市实现地区生产总值9201.16亿元，增长8.2%；财政总收入1920.11亿元，增长10.7%，其中地方一般公共预算收入1027.32亿元，增长8.7%；城镇和农村居民人均可支配收入分别达到44632元和23555元，分别增长9.1%和11.1%；城镇登记失业率1.84%，居民消费价格涨幅2.0%；节能减排指标超额完成省下达任务。

　　过去一年，面对外部复杂环境和改革发展繁重任务，我们坚持抓早抓准抓实，深入开展改革创新年、项目推进年、优化服务年活动，积极应对经济下行、企业“资金链、担保链”和房地产市场风险等严峻挑战，妥善处置各类重大事件，实现了经济社会平稳健康发展。我们成功争取国务院同意创建中国（杭州）跨境电子商务综合试验区和国家自主创新示范区、富阳撤市设区、杭州都市经济圈转型升级综合改革试点、国家生态文明先行示范区创建、杭州航空口岸72小时过境免签政策、阿里巴巴在纽交所上市、大运河申请世界文化遗产、2018世界短池游泳锦标赛和世界游泳大会申办权等重大事项；认真贯彻省委常委会专题研究杭州工作会议精神，落实杭州发展定位新要求；推进实施“杭改十条”、“一号工程”、重大基础设施工程、大江东管理体制调整、市区一体化发展等一批重大举措，为杭州科学发展注入了新活力、积蓄了新动能。总之，过去一年是我市改革创新取得突破、经济发展稳中向好、城乡环境较大变化、民生保障持续改善、精神文明创建深入推进、各方面工作取得显著成效的一年。

　　（一）深化改革取得重大进展。“四张清单一张网”改革全面推进。向社会公布了行政权力清单目录，45个市级部门行政权力从14476项精减到4227项，精减幅度达70.8%；富阳试点经验在全省推广。市级部门责任清单和企业投资“负面清单”公布实施；全面开展市级财政专项资金清理整合，形成了资金管理清单；实施项目审批体制重大改革，建成浙江政务服务网杭州平台。重大管理体制改革取得突破。大江东“一个平台、一个主体”管理体制实体运作，健全城西科创产业集聚区管理体制，萧山、余杭与主城区一体化发展意见制定出台。深化机构改革，市场监管体制改革和市级卫生计生、内外贸机构整合改革完成。“信用杭州”建设取得新进展。市场化改革深入推进。出台实施了“亩产倍增”计划、工业用地评价考核体系、差别化供地、阶梯式水价等资源要素市场化配置改革，滨江工商登记制度改革试点经验在全省推广，市场活力进一步激发，全市新增企业登记5.36万家，增长35.1%；新增上市公司7家，累计109家；浙江股交中心新挂牌企业176家，累计354家；“个转企”12756家，累计25870家，实现四年任务两年完成；实施城镇低效用地再开发16042亩。

　　（二）经济质效得到较大提升。经济平稳协调增长。加快项目建设和有效投资，全市固定资产投资完成4952.70亿元，增长16.2%，其中完成重大项目投资1618亿元，增长36.0%；全市民间投资2851.41亿元，增长26.5%。挖掘消费潜力，社会消费品零售总额3838.73亿元，增长8.7%；网络零售额2088.45亿元，增长37.0%。优化外贸综合服务、落实出口新政，出口491.66亿美元，增长9.8%；不含省级公司出口427.68亿美元，增长11.3%。实际利用外资63.35亿美元，增长20.1%；到位内资1054.07亿元，增长22.5%，其中浙商回归项目到位资金520.06亿元。产业转型升级步伐加快。经济结构继续优化，三次产业比重为3.0∶41.9∶55.1。在推动“十大产业”加快发展的基础上，启动实施以发展信息经济、推进智慧应用为主要内容的“一号工程”，大力推进“四换三名”，高新技术产业、战略性新兴产业、装备制造业增加值增速均快于规上工业。全市工业增加值增长8.6%；规模以上工业企业利税1490.68亿元，增长9.5%，其中利润876.28亿元，增长10.5%。

　　淘汰落后产能企业698家。电子商务、信息软件、文化创意、休闲旅游等现代服务业增势强劲，全市服务业增加值5067.90亿元，增长8.5%；粮食功能区和现代农业园区加快建设，农业增加值增长1.8%；区域性金融服务中心和财富管理中心建设稳步推进，存贷款双双突破2万亿元，年末金融机构本外币存款余额24450.51亿元，增长10.3%；本外币贷款余额21316.83亿元，增长10.2%。自主创新能力提升。坚持科技创新，预计研究与试验发展经费支出占地区生产总值比重达到3%；发明专利授权量5559件；新创建国家级孵化器4家，累计达21家，发明专利授权量和国家级孵化器总量居省会城市第一。杭州高新开发区跻身国家高新区综合排名前五位，未来科技城、青山湖科技城、大江东产业集聚区、杭州经济开发区、钱江开发区等重大平台建设加快推进。

　　（三）城乡建设管理扎实推进。城乡规划和大项目引领作用加强。城市总规修改方案完成并上报审批，钱塘江、运河和西湖沿线景观提升规划工作有效推进。编制完成城市防洪排涝、中小学布局、文化设施等专项规划20项。市域重大交通项目规划建设加快，钱江通道、杭长高铁建成，杭黄高铁开工建设，协调推进都市高速（绕城西复线）、临金高速、千黄高速、运河二通道等项目。交通治堵有新成效。坚持“公交优先”、建设公交都市，主城区公交分担率有新提高。地铁建设力度加大，2号线东南段开通运营，4号线首通段建成。城市快速路初步成网，彩虹快速路滨江段、秋石三期高架主线、东湖路—德胜路立交建成通车；萧山机场高速公路改建、文一路地下通道、环城北路地下通道、紫之隧道等重大工程加快建设。平稳实施了小客车总量调控和城区限行政策，完善道路停车、社会资本建设停车场（库）、老居住小区交通综合治理等政策措施，市区交通拥堵状况明显改善。新增停车泊位53283个，其中公共泊位7711个。主城区增扩公共自行车服务点85处、新增3100辆，全市总量达到8.11万辆。城市管理和环境治理有新加强。启动智慧城管，城市管理长效机制进一步完善，清洁度和序化度达95%以上。恢复湖滨路步行街。生活垃圾“三化四分”有序推进，实施厨余垃圾分类减量工程。强势推进“三改一拆”，新改造旧住宅区、旧厂房、城中村2098万平方米，拆除违法建筑2902万平方米。城乡统筹深入推进。实施区县（市）协作项目114个，落实协作资金3.58亿元；城区向县（市）转移产业项目294个，总投资879.61亿元，县（市）发展综合实力明显增强，主要经济指标增幅继续高于全市平均水平。实施联乡结村共建项目1514个，落实帮扶资金1.62亿元，推进经济薄弱村发展和困难农户持续增收，全市城乡居民可支配收入比由上年的1.93∶1缩小到1.89∶1。完成农村住房改造26635户，实施农村困难家庭危房改造1518户，农村人居条件进一步改善。中心镇和美丽乡村建设全面深化，村级股份制改革加快推进，新农村建设不断加强。

　　（四）美丽杭州建设成效明显。生态建设持续发力。城区新增绿地417万平方米。“三江两岸”生态景观保护与建设、淳安县“美丽杭州”实验区建设扎实推进，杭州成为首个通过省级生态市验收地区。“五水共治”攻坚成效显著。落实“河长制”，消灭垃圾河71条460公里，整治黑臭河193条665公里。全面推进小河流治理和农村污水治理，完成农村生活污水治理844个村，受益农户21.28万户。城西污水处理厂建成运营。城市防洪排涝等水利设施建设全面推进。加强钱塘江、苕溪流域水源保护，一、二级饮用水源保护区内22家污染企业全部关停搬迁；闲林水库基本建成，千岛湖配供水工程开工建设。环境执法力度进一步加大，全市立案查处环境违法案件1008件，移送公安案件45件，刑拘64人。大气治理和节能减排力度加大。大力推进无燃煤区建设、工业污染治理、汽车油品提升、新能源汽车推广和建筑施工扬尘治理，淘汰黄标车和老旧车9.5万辆，市区空气质量有新改善，全年优良天数同比增加13天，PM2.5年均浓度同比下降7.7%。全市关停淘汰重污染高耗能企业298家、整治提升企业425家。杭钢转型升级、半山电厂燃煤机组关停有序推进，全面开展合同能源管理和社会节能工作，全市单位生产总值能耗下降6.4%以上，规上工业单位增加值能耗下降7.9%。节能减排财政政策综合示范工作提前一年完成“十二五”目标。

　　（五）社会民生事业持续发展。民生投入和办实事力度继续加大。全市一般公共预算民生支出达712.58亿元，占一般公共预算总支出的74.1%。全面完成或超额完成了持续改善市民出行条件、加强雾霾治理、提升饮用水质量、提升城市河道水质、加强食品药品安全监管、提高社会保障水平、推进养老助残服务、丰富城乡文体生活、优化小区居住环境、建立便民E邮站等十件实事37个项目。保障性安居工程项目开工36534套，竣工36756套。新增城镇就业24.82万人，城乡基本养老、医疗保险参保率分别达97.07%和98.94%。新增机构养老床位7487张，改扩建社区居家养老服务照料中心610家。提升改造150处社区便民服务点，主城区建成并投入运营889个便民“E邮站”。持社会保障卡人数达821万。社会事业持续发展。各级各类教育统筹协调发展，教育国际化行动计划启动实施，学校体育场地向社会全面开放。智慧医疗便民惠民效果明显，公立医院改革、医养护一体化有序推进。平稳实施“单独两孩”政策。被列为全国首批养老服务综合改革试点城市。积极推进文化名城强市建设，中国动漫博物馆开工兴建，浙江音乐学院校区加快建设。成为“双世遗”城市，国家非遗项目入选数和总量居全国同类城市第一。入选全国精神文明建设“五个一工程”作品4部。高标准建成150个农村文化礼堂，并对已建成的195个进行全面提升。我市运动员在亚运会、青奥会等重大体育比赛中取得好成绩，全民健身运动蓬勃开展。社会治理创新稳步推进。社区建设进一步加强，有效推进“社区减负”工作，成功创建全国和谐示范社区建设示范城市。加强生产安全、消防安全等监管，安全生产主要指标连续11年实现双下降, 事故起数、死亡人数分别下降4.3%和11.3%。加强社会治安综合治理，有效应对“7•5”公交纵火案等突发事件，全年命案破案率100%，平安法治建设取得新成效。

　　（六）政府自身建设有新加强。以政府自身改革为突破口，深入开展服务企业、服务基层活动，提升政府治理和服务管理水平。自觉接受人大法律监督和政协民主监督，主动接受司法、舆论和群众监督。加强地方立法，提请市人大常委会审议的地方性法规7件。建立政府法律顾问制度，首批选聘10名。全面办结702件市人大代表建议，502件市政协建议案、提案。深化群众路线教育实践活动，严格落实中央八项规定和“四风”问题整改，强化重点领域重要环节监督审计，廉政建设持续加强。全市各类会议、活动分别减少16.0%和35.5%。“三公”经费下降37.1%，节约的经费全部用于“五水共治”等民生事业。

　　过去一年，我们扎实推进国防、民兵预备役、人防、打私、防震减灾、新闻出版、民族宗教、慈善救助、机关事务等工作，积极支持工会、共青团、妇联、侨联、残联、科协等开展工作。

　　各位代表，过去一年的成绩来之不易，这是党中央、国务院亲切关怀，省委、省政府和市委正确领导的结果，是全市上下共同努力、团结奋斗的结果。在此，我代表市人民政府，向全市人民和外来建设者，向各位人大代表、政协委员，向各民主党派、工商联、无党派人士，向各人民团体和社会各界人士，向中央驻杭单位和省级各部门，向驻杭解放军和武警部队官兵，向关心支持杭州发展的港澳台同胞、海外华人华侨和国际友人，表示衷心的感谢！

　　各位代表，我们清醒地认识到，全市经济社会发展中还存在不少问题，政府工作还有很多不足。尤其是保持经济平稳较快增长面临较大压力，去年社会消费品零售总额增幅与预期目标有一定差距，发展动力有所减弱；产业结构性问题较为突出，转型升级、节能减排的任务艰巨；重大改革虽有破题，但落地见效仍需不懈努力；城市治理压力加大，交通拥堵、空气不良、水质污染等突出问题尚未根本解决，公共安全、食品安全等方面还有不少薄弱环节；城乡居民持续增收还有不少困难；政府职能转变有待深化，一些政府工作人员改革意识、法治意识、责任担当意识不强，“四风”特别是办事效率不高问题在一些部门和地方仍然存在。对此，我们一定高度重视，采取有力措施，认真改进和解决。

　　二、2015年发展目标和重点工作

　　2015年是全面深化改革的关键之年，是全面深化法治杭州建设的开局之年，也是全面完成“十二五”目标任务、深入谋划“十三五”规划之年。今年工作的指导思想是：认真学习贯彻党的十八大、十八届三中、四中全会精神和习近平总书记系列重要讲话精神，深入实施省委“八八战略”，全面落实省委、省政府和市委决策部署，主动适应发展新常态，坚持以提高经济发展质量和效益为中心，深化改革开放，突出创新驱动，加强城市治理，强化区域协调，改善生态环境，加强民生保障，促进全市经济持续健康发展与社会和谐稳定，努力完成“十二五”规划目标任务，为建设东方品质之城、幸福和谐杭州打下坚实基础。

　　今年经济社会发展的主要预期目标是：地区生产总值增长8%左右；地方一般公共预算收入增长7.5%左右；城镇和农村居民人均可支配收入分别增长8.5%和9.5%左右；城镇登记失业率4%以内，居民消费价格涨幅3%左右；节能减排相关约束性指标完成省下达的目标任务。

　　做好今年工作，要深刻认识新常态下速度变化、结构优化、动力转换的本质特征，全面把握杭州在高起点上新发展的关键环节。必须走在前列勇争先，深入开展“改革开放学上海、转型升级学深圳、美丽建设学天津”活动，树立标杆、奋力赶超，确保在全省更好发挥龙头领跑示范带动作用，确保继续走在全国重要城市前列；必须抢抓机遇增优势，主动对接多重国家战略，深入推进杭州都市圈建设，全力创建中国（杭州）跨境电子商务综合试验区、国家自主创新示范区，抢抓发展先机，提升杭州发展战略地位和优势；必须改革创新求突破，坚持质量、效益、结构优先导向，以深化改革统领体制机制和制度建设，以创新驱动加快经济转型升级，不断激发市场活力、增强内生动力、释放全社会创新创业潜力，增强城市可持续发展能力；必须以人为本促和谐，坚决守住守好民生保障改善、生态环境保护、社会和谐稳定三条“底线”，不断提升百姓富裕、环境优美和社会文明程度，让全市人民共建共享品质生活；必须整体协同强统筹，坚持全市一盘棋，加强市域统筹发展，加强分层分类指导，有效调动各级各层面积极性，精准发力，增强协同创新力；必须强化责任抓落实，坚持负责敢当、优化服务、提升效能，做到工作项目化，细化线路图、时间表和责任制，抓重点克难点，把各项工作落到实处，为“十三五”开局打下扎实基础。

　　为实现今年目标，着力抓好以下重点工作：

　　（一）深化改革开放，不断增强发展活力。科学编制“十三五”规划。加强对事关中长期发展战略全局的重点难点和关键问题研究，深入谋划好重大工程、重大项目、重大政策，科学编制“十三五”规划纲要及专项规划，为实现更长时间、更高水平、更好质量发展打下扎实基础。深化政府自身改革。继续深化“四张清单一张网”改革，建立健全“四张清单”动态调整机制，提升政务服务网功能，加快电子政务体系建设，提高行政效能，推进政府治理体系和治理能力现代化。深化投资审批制度改革，加强行政权力“接、放、管”有效衔接，健全事中事后监管制度。完善全口径政府预算体系，深化财政专项资金管理改革，推进科技投融资体制改革，改进产业扶持方式。深化管理体制改革。全面落实萧山、余杭与主城区一体化发展各项具体措施。推动大江东管理新体制有效运转。深化中心镇改革和小城市培育试点。深化财政管理体制改革、城市管理体制改革、重大项目分级分类分层建设管理体制改革。探索实施大交通管理体制改革、出租车管理体制改革。加快完善国有文化资产管理体制。加快以居住证、积分制为重点的户籍制度改革。深化市场化改革。以要素市场化配置改革为重点，深化投融资体制改革、地方金融创新改革、国资国企改革。适时推进天然气价格改革，完善大公交价费政策，研究环卫有偿服务收费机制。加快农村产权制度改革，持续实施村级集体经济股份制改革，推动农村土地多种形式流转，完善农村宅基地规范化管理机制，加快耕地承包权权证完善和宅基地、农民住房确权登记发证工作。全方位扩大开放。统筹国际国内两个市场和两种资源，统筹对外开放与对内开放，坚持引进来与走出去相结合，积极融入“一带一路”、长江经济带等国家战略，构建开放型经济新体系。支持企业开展多种形式的对外投资和跨国经营，支持企业到中西部和海外建立生产及营销基地，输出产能、技术和管理。支持企业加强先进技术的引进和关键装备的进口。大力发展服务贸易和服务外包。落实招商引资新机制，突出产业招商，加大精准招商力度，引进外资68.5亿美元，引进内资1200亿元，其中浙商回归引进项目到位资金600亿元，使杭州继续在扩大开放上保持领先优势。

　　（二）坚持创新驱动，不断强化发展支撑。加快“两区”创建和创新载体建设。建立中国（杭州）跨境电子商务综合试验区“六体系两平台”，推进国家自主创新示范区“一区多园”建设，力争“两区”尽早获批，充分发挥叠加效应。加强各类高新区、科技城建设，强化创新孵化功能，打造创新发展新引擎。实施省、市两级特色小镇培育计划，大力推进云栖小镇、山南基金小镇、梦想小镇等一批特色小镇建设，拓展众创空间，推进优势特色产业集聚发展，促进产城融合。加强产业平台整合提升。实施各类开发区整合优化提升计划，优化各类开发区的功能布局、产业定位、亩均产出标准、节能减排标准，推动各开发区高端化、特色化、集约化、生态化发展，再造产业发展新平台。强化土地节约集约利用，完成低效用地再开发面积2.8万亩，消化批而未供、供而未用土地4万亩，拓展发展空间。工业要进一步向园区集中，其他区域原则上不再安排工业用地。大力培育发展创新主体。强化协同创新，鼓励企业加大创新投入，推进产品创新、品牌创新、商业模式创新和管理创新。深入实施瞪羚、雏鹰、青蓝、蒲公英计划，大力扶持科技型成长型中小企业发展。研究与试验发展经费支出占地区生产总值比重3%以上。大力实施质量强市、品牌强市、标准强市战略，加快培育一批龙头企业、品牌企业和高新技术企业，持续推进“个转企、小升规、规改股、股上市”，支持企业兼并重组，提升经济竞争力。加强创新机制建设。深化与名院名校名企战略合作，加强国际技术合作，加快建设科技企业孵化器和加速器，鼓励网上技术市场发展，建立标准技术产业服务平台，推动创新成果转化应用。加强国家知识产权示范城市建设，深化科技与金融的结合。全面实施“人才政策27条”，加强人才引进和培育，加强人才与资本的对接，广聚天下英才来杭创业创新，力争到2020年全市人才总量达到250万人，使杭州成为大众创业、万众创新的乐园。

　　（三）加快转型升级，努力提升经济质效。优化投资消费出口结构。聚焦大项目、好项目，加大对基础设施、产业项目、工业技改、生态环保等重点领域的有效投入，全市固定资产投资增长12%左右。适应消费需求和消费方式新变化，培育消费新热点，推进线上与线下、城市与农村消费融合发展，社会消费品零售总额增长11%左右，网络零售增长20%左右。培育出口新优势，拓展新兴市场，发挥一达通、融易通等综合性服务平台作用，实现出口总额增长8%左右。强势推进“一号工程”。以打造万亿级信息产业集群为目标，以智慧产业化和智慧应用为重点，实施信息经济“六大中心”建设和智慧应用三年行动计划，全力推进国际电子商务中心、全国云计算和大数据产业中心、物联网产业中心、互联网金融创新中心、智慧物流中心、数字内容产业中心和中国软件名城建设。加快发展新兴特色产业。对接省“七大产业”发展规划，加快电子商务、文化创意、云计算和大数据、信息、软件、物联网、环保、互联网金融、健康、旅游、时尚、生物医药、高端装备制造、新能源汽车等特色主导产业发展。加大政策要素倾斜和龙头企业培育力度，整合市级产业基金，推进产业集聚，加快推进区域性金融服务中心和财富管理中心建设，形成杭州创新型产业新优势。加快改造提升优势传统产业。优势传统产业、实体经济是我市经济发展的基础，必须坚持工业化和信息化深度融合，加大结构调整力度，提升改造纺织化纤、食品饮料、机械制造、精细化工等优势传统产业，保护历史经典产业，做大做强“老字号”企业。深入实施“四换三名”工程，加快利用文化创意、物联网、智能制造改造提升传统产业，发展新兴服务业，加快商品房“去库存”化，推进产业跨界融合，培育经济新增长点，打造杭州经济升级版。加快发展现代农业。大力发展生态农业、设施农业、都市农业、智慧农业，建设绿色农业强市。加快粮食功能区、现代农业园区和城市保供基地建设，新建10万亩粮食生产功能区，完成粮食种植面积193万亩，保障粮食安全；新建省级现代农业园区6个，全市新增蔬菜直供直销量1.5万吨以上。推进科技兴农，促进农业增效。积极发展乡村休闲旅游、现代民宿、农村淘宝、养老养生等新业态，使农民增收成为新常态。

　　（四）加强城市治理，提升城市功能和品质。着力完善城市功能布局。开展市域城乡发展战略研究，推进经济社会发展规划、城市总体规划、土地利用规划、生态环境保护规划等“多规融合”，进一步优化空间资源配置。严格实施修改后的城市总规，加强重要地区城市设计，强化城市设计对塑造城市风貌的引导。加快重点区域建设进度，完善提升主城、副城、组团的基础设施和公共服务功能，深化地下空间开发利用，促进城市功能转型提升。大力提升城市国际化水平。全面实施《加快推进城市国际化行动纲要》。提高萧山国际机场口岸国际化程度，新辟欧美城市航线，完善东亚和东南亚航线网络。加快国际博览中心、奥体中心建设，承接国际性会议展览和体育赛事，办好中国国际动漫节、西湖国际博览会、文博会等展会，提升国际影响力。推进人才国际化，加快国际化学校、国际化医院等配套设施建设，增强国际化服务能力。打好交通治堵攻坚战。启动城市快速轨道交通三期建设规划编制，加快2号线西北段，4号线南段和5号线、6号线一期等地铁工程建设，建成1号线下沙延伸段。加快文一路地下通道、紫之隧道、萧山机场高速公路、望江路过江隧道等工程建设，建成环城北路地下通道、秋石四期高架等快速通道，完善“四纵五横”城市快速路网体系。打通断头路，建设与整治支小路，完善公共交通换乘枢纽、停车场站、接驳系统等配套设施建设。完善小汽车总量调控管理办法，提高综合交通管理智能化水平，加快公交都市建设。高效能推进城市管理。以城中村改造为重点，深化“三改一拆”，实施“创无违建县（市、区）三年行动计划”，开展存量违法建筑“清零行动”。加强建筑形态整治，优化建筑立面形态。开展对入城口、城乡结合部、地下通道等重点部位整治，加强城市地下管线和广告管理，做好城市历史文化保护，推进西湖、运河、钱塘江等“三线”和主要商业街道、中心广场的景观与灯光提升工作。深入推进城市管理智慧化、网格化和管理全覆盖，消除盲点、攻克难点，严格落实“五化”长效管理措施，让杭州成为名副其实的国内最清洁城市。

　　（五）加强区域统筹，提升城乡一体化发展水平。积极推动城市群和都市圈合作发展。加强与上海等长三角城市的对接合作，深化杭州都市圈合作发展，深入实施《杭州都市经济圈发展规划》和《杭州都市区规划》，提升杭州中心城市集聚和辐射能级。研究杭州市域统筹发展、九城区协调发展新机制，加快推动萧山、余杭与主城区一体化发展，全面启动富阳融入主城区工作，加快县（市）中心城区建设。加强区域重大基础设施规划建设。加快杭黄高铁等重点项目建设，加快推进千黄高速、临金高速、都市高速（绕城西复线）等重点工程。加强都市圈轨道交通体系规划，加快推进市区连接富阳、临安等周边区县（市）城际轨道建设。实施市区西部交通畅通“三年行动计划”，完善市域交通网络体系。推进城市信息化，加强新一代移动通信网和下一代互联网建设，实现宽带无线网络深度全覆盖。加强供电、供水、燃气和垃圾处理等公用设施建设，增强城市安全保障能力。加强城乡统筹协作发展。深化完善区县（市）协作，持续推进“六大西进”行动，促进临安、桐庐、建德、淳安县（市）与主城区协调发展，增强发展后劲，提升发展水平。加强公共服务基础设施建设，提升农村公共服务水平，推进城市管理向乡镇延伸。深入实施“联乡结村”帮扶工作，积极扶持经济薄弱村集体经济发展。加快中心镇和美丽乡村建设。加强省级小城市培育试点，深化中心镇“双千工程”建设，提升中心镇综合承载力。创建“杭派民居”示范村，推进历史文化村落保护利用，因地制宜深化新农村建设，打造美丽乡村升级版，让乡村更好地保留特色风貌、留得住青山绿水、记得住乡愁。

　　（六）坚持生态优先，深化美丽杭州建设。以更大力度实施环境“四治”。我们宁可少建一栋大楼，也要加大环境治理投入力度。加大治水力度，实施“消灭黑臭河和劣V类水三年行动计划”，重点治理和睦港等城郊接合部黑臭河道、农村污水；建设之江污水处理厂，全面实施七格等城镇污水处理厂提标改造；建成投运三堡排涝、钱江水利枢纽等重点水利工程，加强中小河流治理，提高区域排涝能力；完成闲林水库等主体工程，全面推进千岛湖配供水工程建设；继续实施高层住宅二次供水设施改造，推进雨水和河道水利用。加大治气力度，实施燃煤热电企业清洁排放技术改造和高污染锅炉改造，加快杭钢转型升级、半山和萧山电厂燃煤机组关停工作，基本完成城市建成区无燃煤区建设；实施西湖景区机动车环保行动；加强道路和工地扬尘治理。加大治废力度，深化生活垃圾“三化四分”，加强渣土管理和建筑垃圾综合利用，加强污泥处理和有效利用，推进九峰环境能源项目、绿能发电等垃圾焚烧设施建设和提标改造。加大治土力度，落实农业土壤污染防治三年行动计划，改善提升土壤质量。以更高标准加强生态保护。深化国家生态市创建、生态文明先行示范区建设试点、淳安县“美丽杭州”实验区建设，扎实推进美丽县（市、区）建设。完成环境功能区划分，强化生态红线管控，严格落实环境准入制度，健全流域生态补偿机制，实施新一轮“四边三化”整治工程，加大生态保护力度。提升西湖、“三江两岸”、运河沿线生态景观，加快构建多层次多功能覆盖城乡的基本生态网络。以更大决心淘汰落后产能。推进工业园区生态化改造，加大对污染企业和落后产能的整治淘汰力度，强化节能减排硬约束。扩大工业锅炉运行远程在线监测和能效检测，加强环境检测，鼓励低碳循环发展。支持新能源汽车推广应用。落实生态环境财政奖惩制度。加强行业整治、污染源治理和面源污染防治，打好重污染高能耗行业整治收官之战。以更严要求加强环境执法。严格执行新修订的《环境保护法》，不怕得罪人，坚决以铁的手腕推进环境监管和执法，严厉打击环境违法犯罪，打造环境监管最严格城市，让人民群众在天蓝、水清、地净的良好生态环境中生产生活。

　　（七）坚持惠民安民，持续增进人民福祉。做好就业创业和居民增收工作。重点做好高校毕业生、就业困难人员、社区矫正人员等群体的就业创业服务工作。实施精准扶贫，适时提高城乡居民最低生活保障标准，推动农民和低收入群体持续增收。进一步加强社会保障。完善城乡统筹社保模式，加快推进市区社保一体化。加快养老服务体系建设，推进公办养老机构改革，重点发展社区居家养老。推动住房保障向“以货币为主、实物为辅”转变。大力推进社会事业发展。组织实施“十大教育行动”，重点落实住宅小区配套教育设施项目建设。加强现代职业教育体系建设。推进杭师大二期工程及市属高校项目建设，建成启用浙江音乐学院。深化公立医院改革，实施院前急救条例，鼓励社会力量办医，继续做好智慧医疗、医养护一体化、国家卫生城市复评、健康城市建设等工作，继续做好计划生育工作。加快体育强市建设，认真开展2018世界短池游泳锦标赛等大型赛事筹备工作。加强各级档案馆、农村文化礼堂和城市文化公园建设，推进基本公共文化服务标准化、均等化。加强社会主义精神文明建设。大力培育和践行社会主义核心价值观，健全发现“最美现象”、弘扬“最美精神”长效机制，持续提升市民文明素质。加强和创新社会治理。加大社会组织培育发展力度，提高城市治理的社会参与度。推进桐庐县基层社会治理机制创新改革试点。实施重大决策社会稳定风险评估，完善突发事件应急处置机制。加强反恐维稳工作，推进立体化社会治安防控体系建设，深入开展群防群治专项活动，完善人民调解机制，提高网络、交通、消防等重点领域治理能力。强化安全生产责任，加强重点活动、节假日、人员密集场所等公共安全管理，强化食品药品和农产品质量安全监管。继续做好国防动员、双拥、人防等工作，加强民族宗教、统计、气象防震减灾、对台、老龄、妇女儿童、残疾人、慈善救助等工作，让人民群众拥有更加安居乐业的环境。

　　各位代表，办好民生实事，是我市长期坚持的重要制度，也是政府工作的出发点和落脚点。今年，我们按照群众提、群众定的理念，明确了十个方面的民生实事，我们将全力把民生实事办实办好。

　　一是促进社会就业创业。新增城镇就业22.91万人、城镇失业人员再就业10.72万人。新增大学生创业企业1200家，带动就业6000人以上，组织大学生实训和见习训练6万名。二是加大水环境治理力度。完成市区黑臭河道治理30条（段）37.6公里。完成截污纳管项目300个，4.5万吨/日截污量。完成城市河道清淤30条（段）30公里。市区治理积水项目100个。完成农村河道综合整治300公里。完成农村生活污水治理623个村，受益农户19万户以上。完成山塘水库除险加固124座、中小河流重要堤防加固89公里。改造水利排灌渠道350公里，新增旱涝保收面积6万亩，改善灌溉面积2.8万亩。三是持续改善交通出行。开通运营地铁4号线首通段、1号线下沙延伸段。新辟10条、优化30条公交线路。新增（扩容）公共自行车服务点100处，新增（更新）公共自行车3000辆。新建成汽车停车泊位46000个，其中新增公共停车泊位6000个。完成农村公路大中修450公里。四是强化食品安全治理。加强无证照餐饮整治，固定场所无证无照餐饮整治率达90%以上；实施量化分级管理，大中型以上餐饮企业实施率100%，持证小餐饮实施率85%以上。提升学校食堂餐饮量化分级，学校A、B等级达80%，学校午餐配送单位“阳光厨房”建设率达80%。食品定量检测达5批次/千人。新创建放心农贸市场38家。五是加强养老为老服务。新增养老机构床位5000张，新改扩建城乡社区居家养老服务照料中心500家。依托社区公共服务平台搭建市级养老服务综合信息平台，新发放智慧养老服务终端2万台。为杭州户籍60周岁以上享受城乡最低生活保障待遇人员、城镇“三无”人员、农村“五保”及重点优抚对象、60周岁以上失独人员、80周岁以上高龄老人购买意外伤害保险。六是加大雾霾治理力度。完成全市淘汰黄标车目标；推广应用新能源汽车2500辆；推进重点行业挥发性有机物治理，完成30家以上重点企业治理任务，年削减挥发性有机物排放1000吨以上；开展加油站、油罐车及储油库的油气回收工作，回收450吨以上；加强建筑工地扬尘治理，创建文明施工示范工地120个以上。七是改善城乡人居环境。完成公共租赁住房（廉租住房）配租房源4000套，公开销售经济适用住房10万平方米。完成21996户农村住房改造、1460户农村困难家庭危房改造。推进农村饮水安全提升工程，改善和提升10.5万人。消除或增亮街巷和游步道1000处暗区。新增管道燃气用户5万户。新增城市绿地260万平方米。八是丰富城乡文体生活。建设农村文化礼堂100个。为100个城乡社区提供图书、演出、电影、辅导、器材等文化服务，为社区群众展示特色才艺提供平台。新建健身公园、健身广场、健身中心等城乡公共体育设施20处，新增社会体育指导员1000名。九是加快电商服务网络建设。继续推广建设社区E邮站快递包裹智能存取服务平台，主城区新建站点800个以上；新增农村电子商务乡村服务站（点）500个以上。十是加强法律援助和服务。扩大法律援助范围，深化律师进社区活动，新增法律援助站点108个，受理法律援助案件1.2万件，举办法律讲座250场以上，为中小学生和外来务工人员提供义务普法，为10万人提供免费法律咨询服务。

　　三、全面推进法治政府建设

　　全面贯彻依法治国基本方略，加快建设职能科学、职权法定、执法严明、公开公正、廉洁高效、守法诚信的法治政府，加快建设人民满意的服务型政府。

　　（一）自觉带头学法守法用法。深入学习中央和省市委全面推进依法治国、深化法治浙江和法治杭州建设等重大决策部署。落实政府常务会议学法制度和法律讲座制度，加强政府法制建设，引导全民学法，加快建设法治社会。加强公务员队伍培训和管理，强化公务员法治意识，提高运用法治思维和方式解决实际问题能力，全面提高公务员的政治理论水平和业务素质。

　　（二）依法规范和履行政府职责。坚持法无授权不可为、法定职责必须为。修订完善市直部门主要职责、内设机构和人员编制“三定”规定，推进区县（市）政府和部门机构、职能、权限、程序、责任的法定化和政府各项事权规范化法律化，构建高效的政务生态系统。严格落实执法责任制，强化重点领域执法，推进综合执法。推进政府服务信息化、精细化和高效化，提高政府管理科学化、绩效化水平。

　　（三）健全依法决策机制。围绕信息经济、城市治理、生态保护、平安杭州等重点领域开展立法，清理和修改规范性文件。出台《杭州市人民政府重大行政决策程序规则》，完善重大决策和规范性文件合法性审查制度，提高政府科学立法和规范性文件管理水平。健全重大决策后评估和纠错制度、责任追究制度和倒查机制，提升科学民主决策水平。

　　（四）强化对行政权力的制约和监督。认真执行市人大及其常委会决议，坚持重大事项报告制度。自觉接受人大法律监督、政协民主监督，主动接受司法监督、社会监督和舆论监督，加强审计监督，优化综合考评，规范行政行为。深化政务公开，以财政预决算、建设项目、社会公益事业等领域政府信息公开为重点，推进行政决策、执行、管理、服务和结果公开。加强“信用杭州”建设，完善“公述民评”、“杭州发布”等载体，营造公开公正公平的发展环境。

　　（五）创新政府管理服务方式。加大简政放权力度，处理好政府与社会的关系，进一步向区县（市）和乡镇（街道）放权。切实提高办事效率。健全公共资源市场化配置机制，积极推进政府购买公共服务的标准化和规范化，逐步将行业管理、社会生活服务管理职能转移给有资质的社会组织，加强对社会中介组织的监督和指导，健全后续监管机制和评价体系。

　　（六）加强作风建设和廉政建设。巩固群众路线教育实践活动成果，防止“四风”反弹，认真开展“三严三实”专题教育活动，严守政治纪律和政治规矩，做到忠诚为民务实清廉。着力解决政府工作人员“不作为”问题，形成勇于负责、敢于担当的优良作风。加强对财政资金、机关事业单位办公用房及相关资产的使用管理，厉行节约，反对浪费，严控“三公”经费支出。坚定不移推进廉政建设和反腐败斗争，加强反腐倡廉制度建设，突出抓好工程建设、土地市场、房地产开发等重点领域和招标投标、设计变更、政府采购、公款存储等重点环节的监管力度，从源头上防治腐败。

　　各位代表，新的目标激励人心、新的任务艰巨光荣、新的征程催人奋进，让我们紧密团结在以习近平同志为核心的党中央周围，在省委、省政府和市委的坚强领导下，以更加饱满的热情、更加昂扬的斗志、更加务实的作风，锐意进取、扎实工作，为建设东方品质之城、幸福和谐杭州而努力奋斗！
